# Laminarin Alleviates Acute Lung Injury Induced by LPS Through Inhibition of M1 Macrophage Polarisation

**DOI:** 10.1111/jcmm.70440

**Published:** 2025-03-05

**Authors:** Liming Zeng, Jieyu Zhang, Rongrong Song, Xinhuai Dong, Zibo Wei, Xiaoyan Li, Xiaokang Zeng, Jie Yao

**Affiliations:** ^1^ Medical Research Center & Department of Laboratory Medicine of Shunde Hospital Southern Medical University (The First People's Hospital of Shunde) Foshan Guangdong China; ^2^ Central Laboratory of The Sixth Affliated Hospital, School of Medicine South China University of Technology Foshan Guangdong China; ^3^ Clinical Laboratory of Shunde Hospital Southern Medical University (The First People's Hospital of Shunde) Foshan Guangdong China; ^4^ Clinical Laboratory of The Sixth Affiliated Hospital, School of Medicine South China University of Technology Foshan Guangdong China

**Keywords:** acute lung injury, laminarin, macrophage polarisation, sepsis

## Abstract

The lipopolysaccharide‐induced acute lung injury (ALI) mouse model is used to simulate human acute respiratory distress syndrome (ARDS), which has a high mortality rate. An imbalance between M1 and M2 macrophages, characterised by an increase in M1 macrophages, was observed in sepsis‐induced ALI. We report that laminarin, an active ingredient found in algae, exhibits exceptional performance in a mouse model of sepsis‐induced ALI. It ameliorates lung edema, enhances the survival rate of mice and reduces the levels of the inflammatory factors TNF‐α and IL‐6. Furthermore, laminarin reduced the expression of CD86, which are markers associated with M1 macrophages. Laminarin treatment reduces the secretion of TNF‐α and IL‐6 in LPS‐stimulated macrophages. Laminarin treatment also decreases glucose uptake in LPS‐stimulated macrophages. Transcriptome sequencing reveals that genes downregulated in LPS‐stimulated macrophages following laminarin treatment are predominantly enriched in the HIF‐1α signalling pathway. Experimental validation confirms that laminarin treatment of LPS‐stimulated macrophages reduces the expression of HIF‐1α and significantly decreases the expression of related indicators ROS and NLRP3. After using siRNA to knock down HIF‐1α in RAW264.7 cells, the inhibitory effect of laminarin on LPS‐induced M1 polarisation of macrophages is abolished. This suggests that laminarin may potentially inhibit macrophage polarisation towards the M1 phenotype by downregulating the HIF‐1α signal. In conclusion, the data presented in our study demonstrate that laminarin can effectively reduce M1 macrophage polarisation by downregulating HIF‐1α signalling. This makes it a novel candidate drug for the treatment of LPS‐induced ALI.

## 1 Introduction

Sepsis typically arises from severe infections, trauma, shock and burns and has emerged as a common complication during the COVID‐19 pandemic [1]. Sepsis can lead to a variety of complications, with Acute Respiratory Distress Syndrome (ARDS) being one of the most common [2], and it significantly impacts the incidence and mortality rates of patients with sepsis [3]. Acute Lung Injury (ALI) often progresses to ARDS, which is characterised by an excessive and uncontrolled inflammatory response to lung injury, leading to systemic epithelial and endothelial barrier damage, dysfunction of the alveolar‐capillary membrane, increased vascular permeability, alveolar haemorrhage and diffuse alveolar damage. Clinical manifestations include severe hypoxemia, diffuse bilateral pulmonary infiltrates and pulmonary edema [4, 5, 6]. Presently, no universally effective treatment for ARDS exists worldwide. Corticosteroids are the primary choice, albeit linked with increased infection risk [7]. Hence, there is an urgent need to develop safer and innovative therapies for ARDS induced by sepsis.

Macrophages are an essential component of the innate immune system and are present in various tissues [8]. Macrophage activation is considered the critical initial stage of the inflammatory response, and it can recruit other cells to the site of inflammation and regulate their status [9]. Macrophages have high plasticity and can be polarised towards distinct functional phenotypes depending on the presence of different stimuli [10]. In particular, many studies have found that macrophages are polarised towards the M1 (proinflammatory) phenotype rather than the M2 (anti‐inflammatory/reparative) phenotype in acute lung injury (ALl), leading to aggravation of lung inflammation [11]. In ALI induced by sepsis, there is a disruption in the balance between M1 and M2 macrophages, characterised by a significant increase in M1 macrophages [12]. M1 macrophages can promote and regulate the occurrence of inflammation [13], secreting a large amount of cytokines, such as IL‐1β, inducible nitric oxide synthase (iNOS), tumour necrosis factor‐α (TNF‐α), reactive oxygen species (ROS) and cyclooxygenase 2 (COX‐2), which may trigger a cytokine storm in the early stages of sepsis [14, 15]. In ALI, during the early stages of ALI, macrophages are mostly polarised into M1 macrophages [16], secreting inflammatory mediators. They can help initiate an inflammatory response. Studies [17] have confirmed that in a LPS‐induced acute lung injury model in mice, the number of M1 macrophages in the lungs of the mice increased and was closely related to the severity of ALI.

Marine‐derived laminarin has been widely used due to its bioactivity, safety and low production costs. Laminarin, a low molecular weight β‐glucan storage polysaccharide present in brown algae, can be used to enhance biological activity and is utilised in cancer therapy, drug/gene delivery and has also shown anti‐inflammatory and antioxidant capabilities [18]. Liu et al. confirmed that [19] the use of laminarin leads to a decrease in mRNA encoding M1 macrophages and an increase in mRNA encoding M2 macrophages, indicating that laminarin has the effect of inhibiting the polarisation of macrophages towards the M1 phenotype. Currently, there are numerous reports on the correlation between macrophages and ALI, but the mechanism of using laminarin to inhibit the polarisation of M1 macrophages in ALI to alleviate its occurrence and development is not clear yet.

This study investigates the impact of laminarin on ALI induced by sepsis and its effect on macrophage polarisation, both in vitro and in vivo. Our findings provide initial evidence that laminarin mitigates sepsis‐associated ALI by inhibiting HIF‐1α‐mediated M1 macrophage polarisation.

## 2 Materials and Methods

### 2.1 Protocol Approval

The protocol of the study associated with human specimens was approved by the Ethics Review Committee of Shunde Hospital, Southern Medical University (KYLS20231109), Foshan, Guangdong, China, and all study procedures were performed in accordance with the guidelines of the Declaration of Helsinki. All patients signed a written informed consent form. Our animal experiment protocols were reviewed and approved by the Animal Ethics Committee of Southern Medical University, China (No. SDYY‐YJ‐2205‐002). The animal experiments were implemented in strict accordance with the approved protocols.

### 2.2 PBMCs Isolation

Peripheral blood mononuclear cells (PBMCs) were isolated following these steps: Peripheral blood from patients with sepsis or volunteers was collected and added to an equal volume of Ficoll slowly. The preparation was then centrifuged at 400 *g* for 25 min, and PBMCs were obtained from the middle edge of the preparation. Subsequently, PBMCs were washed with PBS twice. Finally, PBMCs were cultured in 1640 medium (Gibco) with 10% FBS (Gibco).

### 2.3 Cytotoxicity Assays

A CCK‐8 assay was conducted to assess the viability of laminarin on RAW264.7 cells. The procedure was as follows: Cells were initially seeded onto 96‐well plates and cultured in DMEM (Gibco) with 10% FBS overnight. Various concentrations of laminarin (Sigma, L9634) were prepared, including 5, 2.5, 1.25, 0.625, 0.3125, 0.15625, 0.078125, 0.039063 and 0.019531 mg/mL. A control with 0 mg/mL laminarin was also included. The cells were then cultured with the respective laminarin concentrations for either 24 or 48 h. Subsequently, 10 μL of CCK‐8 solution was added to each well, and the cells were further incubated for an additional hour. Finally, the absorbance at 450 nm was measured and recorded to determine cell viability.

### 2.4 Cell Culture

For RAW264.7 cells, the following protocol was followed: 100 ng/mL of lipopolysaccharide (LPS) was added, and the cells were cultured for 24 h. Subsequently, 0.3125 mg/mL of laminarin was added to the cells in DMEM medium containing 10% FBS. After another 24 h, the supernatant was collected for ELISA and nitric oxide (NO) detection. For PBMCs, the procedure was as follows: PBMCs were cultured in RPMI 1640 medium containing 10% FBS. Cells were treated with 0.3125 mg/mL of laminarin or different concentrations of MIF (macrophage migration inhibitory factor) including 100, 50, 25 ng/mL and a control with 0 ng/mL MIF (MCE, HY‐P7387). The cells were cultured for 24 h under these conditions.

### 2.5 ROS Level Detection

RAW264.7 cells were harvested and washed with PBS twice and then 1 mL oxidation‐sensitive fluorescent probe (DCFH‐DA, obtained from Beyotime, diluted with DMEM at 1:1000) was added, and the mixture was incubated at 37°C for 20 min; flow cytometry was used to detect the level of ROS. The expression level of ROS is expressed as percentage.

### 2.6 NO Level Detection

Griess Reagent (Beyotime) was used to detect the release of NO. 50 μL cell supernatant was added to a 96‐well plate; then, 50 μL Griess Reagent I and Griess Reagent II was added in order; absorbance at 520 nm was detected and recorded. Finally, the concentration of nitric oxide in the sample was calculated according to the standard curve.

### 2.7 Detection of Lactate Release

Lactate in supernatant was detected using the Lactate Colorimetric Assay Kit II (BioVision). Briefly, 50 μL/well of sample was added to a 96‐well plate; next, 50 μL of the Reaction Mix (prepared according to the instructions for manufacture) was added to each well, and the mixture was incubated for 30 min at room temperature. Finally, absorbance was detected at 450 nm, and lactate concentrations were calculated according to the standard curve.

### 2.8 Glucose Concentration in RAW264.7

Glucose concentration in RAW264.7 was detected by the Glucose Assay Kit‐WST (Dojindo). The operation steps were followed according to the instructions from the manufacturer, and absorbance at 450 nm was detected and recorded. The concentrations of glucose were calculated according to the standard curve.

### 2.9 Cytokine Assays

Detection of IL‐6 from the serum of mice and the supernatant of RAW264.7 was carried out by the Mouse IL‐6 Precoated ELISA kit (Dakewe Bio‐engineering Co., LTD) according to the manufacturer's instructions.

### 2.10 Flow Cytometry

For flow cytometry, cells were washed with cold PBS containing 3% FBS. Then, for RAW264.7, FITC‐conjugated anti‐mouse CD68 antibody (Biolegend, #137006), PE‐conjugated anti‐mouse CD86 antibody (Biolegend, #137014), for cells isolated from lungs, FITC‐conjugated anti‐mouse CD86 antibody (BD, #561962), PE‐conjugated anti‐mouse CD68 antibody (BD, #566387), FITC‐conjugated anti‐mouse CD86 antibody (Invitrogen, #53‐0869‐42), FITC‐conjugated anti‐mouse Ly6G antibody (ThermoFisher, 1A8‐Ly6g) and eFluor 450‐conjugated anti‐mouse CD11b antibody (ThermoFisher, M1/70) were added, and cells were incubated at 4°C for 1 h. After washing twice, the level of CD86 expression was detected by BD flow cytometry. MFI or percentage (%) was considered as expression levels. Data were analysed by Flowjo software.

### 2.11 Western Blot

Cells were washed twice and lysed with PIRA lysis containing 1% PMSF; for lungs from mice, after washing with PBS, the lungs were homogenised and lysed. Subsequently, total protein was obtained, and the concentration of which was detected by BCA assays. After boiling with SDS‐PAGE loading buffer for 10 min, the protein samples were separated by electrophoresis and transferred to PVDF membranes. Then, the PVDF membranes were blocked with 5% BSA and incubated with anti‐HIF‐1α antibody (1:1000, Abcam, #ab179483) and anti‐eNOS antibody (1:1000, Abcam, #ab5589) at 4°C overnight. The next day, PVDF membranes were incubated with HRP‐conjugated IgG secondary antibodies (1:1000, Beyotime) for 1 h. Finally, bands were visualised by ECL Western Blot Kit (Millipore), and the absorbance values of protein bands were analysed by Image J software.

### 2.12 RNA Sequence Analysis (scRNA‐Seq)

Total RNA from RAW264.7 was extracted by trizol. Subsequently, RNA sequencing was performed by OE Biotech Co. Ltd. (Shanghai, China) on the Htseq‐count platform. DESeq was used to identify differentially expressed genes (*q* < 0.05 and |log2FC| > 1).

### 2.13 Construction of Sepsis Model in Mice

Male C57BL6/J mice aged 8–12 weeks were obtained from Beijing Hua Fu Kang Biotechnology Co. LTD (Beijing, China). The mice were housed in 12‐h dark/light cycles with the temperature at 26°C ± 2°C, 50% humidity and enough food and water. Mice were divided into three groups randomly: Control group, LPS group and LPS + laminarin group. For the LPS group, mice received 35 mg/kg LPS (i.p.). One hour after being injected with LPS, mice in the LPS + laminarin group received 800 mg/kg laminarin (i.p.), while mice in the control group received the same volume of PBS (i.p.). Six hours after LPS injection, mice were sacrificed, and blood was obtained from the right ventricle orbital veniplex; serum from blood samples was obtained after centrifuging at 3500 rpm for 15 min. Moreover, lung tissue from mice was obtained and washed for assays of wet/dry weight ratio, western blot, flow cytometry, immunohistochemistry and haematoxylin–eosin staining assays.

### 2.14 Assays of Wet/Dry Weight Ratio

Lungs obtained from mice and absorbent paper were used to absorb liquid on the surface of the lungs. Subsequently, the weight of the lungs was recorded and considered as wet weight. After drying at 60°C for 48 h, the lungs were re‐weighted and recorded as dry weight. The degree of lung edema was evaluated by calculating the wet/dry weight ratio.

### 2.15 Immunohistochemistry and Haematoxylin–Eosin Staining Assays

Lungs obtained from mice were fixed in 4% paraformaldehyde and embedded in paraffin. Tissues were sliced into 5 μm slices and deparaffinised, and rehydrated with xylene and graded ethanol solution. Then, 3% H_2_O_2_ and citrate buffer were carried out to inactivate endogenous peroxidase and restore antigens. Next, the sections were blocked with 5% bovine serum albumin (BSA) for 30 min at 37°C. Subsequently, anti‐iNOS antibody (1:2000, Abcam, #ab283655), anti‐Glut1 antibody (1:250, Abcam, #ab115730), anti‐NLRP3 antibody (1:100, Affinity, #DF7438) and anti‐HIF1α antibody (1:100, Affinity, #AF1009) were added and incubated at 4°C overnight. After that, HRP‐conjugated secondary antibodies (1:500, Beyotime) were added and incubated for 30 min at 37°C. The samples were processed with DAB chromogen solution and counterstained with haematoxylin. For haematoxylin–eosin staining assays, sections that had been deparaffinised and rehydrated were stained with haematoxylin, then treated with 1% hydrochloric acid ethanol, followed by eosin. Finally, the samples were detected by microscopy, and images were captured. Data were analysed by Image Pro plus 6.0.

Lung injury scoring system: According to the Official American Thoracic Society Workshop Report [20], we scored 30 random high‐power fields (400× total magnification) to measure lung injury, including A. Neutrophils in the alveolar space; B. Neutrophils in the interstitial space; C. Hyaline membranes; D. Proteinaceous debris filling the airspaces; E. Alveolar septal thickening in five parts, and each field scores as 0, 1 and 2. Finally, Score = [(20 × A) + (14 × B) + (7 × C) + (7 × D) + (2 × E)]/(number of fields × 100).

### 2.16 Small Interfering RNA Transfection

The siRNA sequence targeting HIF‐1α is as follows: CACCATCAGTTATTTACGT. Seed 1–2 × 10^5^ RAW 264.7 cells into each well of 12‐well plates. Once the cells reach 30% confluence, prepare the transfection reagent complex in a centrifuge tube. Sequentially add jetPRIME buffer, siRNA (final concentration 50 nM), and jetPRIME reagent, then vortex mix. Incubate the transfection complex at room temperature for 10 min. Add the transfection reagent complex dropwise to the cells in complete medium without antibiotics. Place the culture plates in a 37°C, 5% CO_2_ incubator for 48 h. After incubation, remove the transfection reagent and wash the cells with PBS. Add 50 ng/mL LPS and/or 0.3125 mg/mL laminarin to the cells and continue to incubate for 24 h.

### 2.17 Statistical Analysis

Data are reported as mean ± standard deviation (SD) and shown by GraphPad Prism software (GraphPad Software Inc., San Diego, CA, USA). Data were analysed statistically by SPSS software. Each experiment was repeated three times unless noted. The differences between groups were calculated using the two‐tailed Student's *t* test or one‐way ANOVA or wo‐tailed Student's *t* test. *p* < 0.05 was considered to be statistically significant. *p < 0.05, **p < 0.01; ***p < 0.001.

## 3 Results

### 3.1 Laminarin Benefits Mice Suffering From Acute Lung Injury Induced by LPS‐Induced Sepsis

**FIGURE 1:**
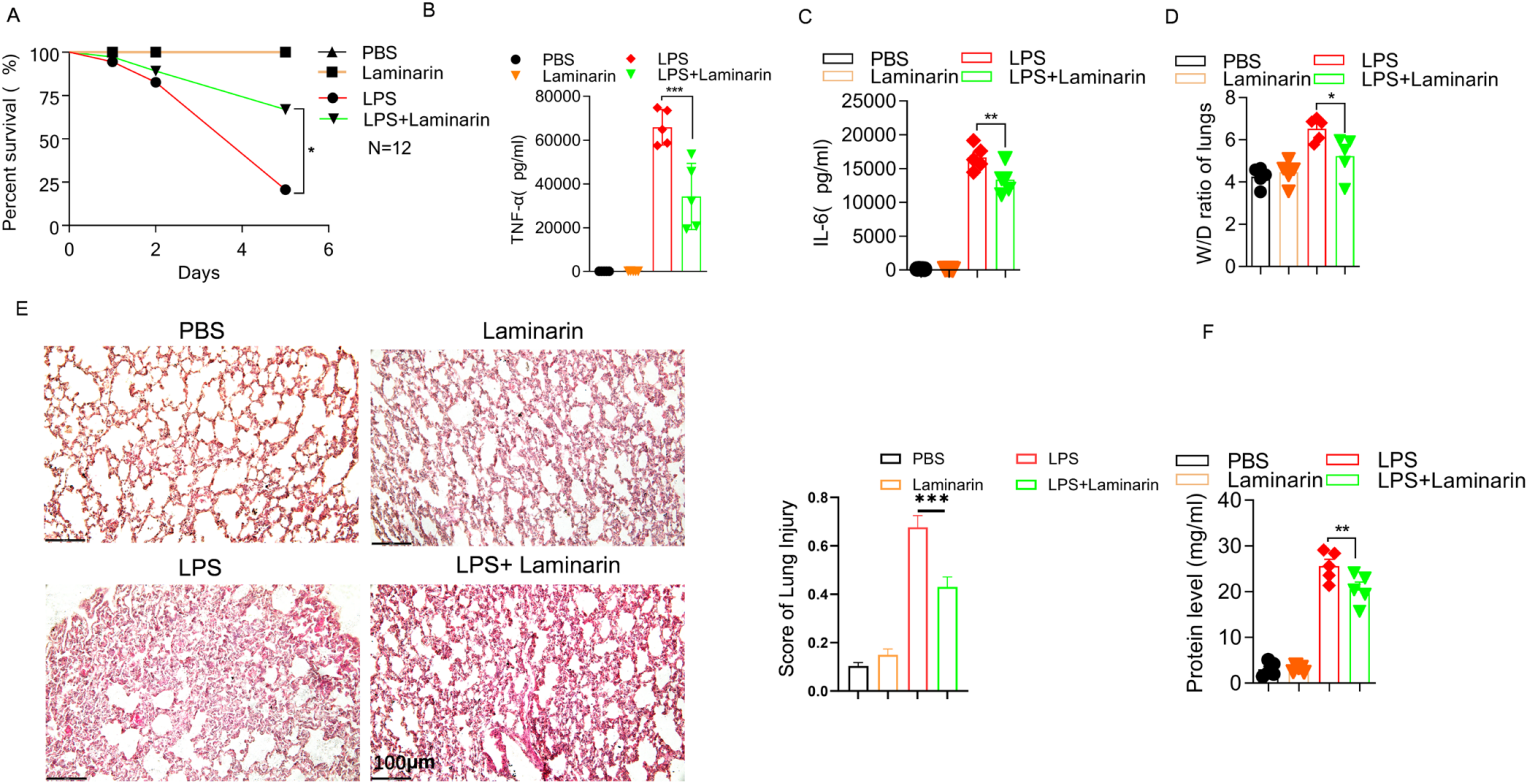
Laminarin alleviates acute lung injury in a mouse model of LPS‐induced sepsis. Male C57BL6/J mice aged 8–12 weeks were randomly separated into 3 groups: Control group, LPS group, and LPS + laminarin group. (A) Percentage of survival was evaluated once a day for 5 days, and the number of deaths in each group was recorded (*n* = 12). (B) Expression level of TNF‐α in serum was analysed by ELISA (*n* = 5). (C) Expression level of IL‐6 in serum was analysed by ELISA (*n* = 5). (D) After 6 h post‐injection of LPS, mice were sacrificed, and lung tissues were harvested. The effect of laminarin on acute lung injury in septic mice was assessed by the wet/dry ratio (W/D) and (E) Haematoxylin–eosin staining (Scale bar magnification = 100 μm), along with the statistical analysis of pathological scoring charts (*n* = 5). (F) Statistical analysis of total protein concentration in the BALF of each group of mice (*n* = 5). Data are presented as mean ± SD and analysed by one‐way ANOVA; *p* < 0.05 was considered a significant difference between the LPS group and LPS + laminarin group; *p < 0.05; **p < 0.01; ***p < 0.001. Data are representative of three independent experiments.

In this study, to investigate the effects of laminarin in the context of sepsis, a mouse sepsis model was established using 35 mg/kg of LPS (Figure S1). Laminarin was administered one hour after LPS injection, and the mortality was monitored. The data indicated that the survival rate of septic mice improved by 49% at 5 h after administration of laminarin (Figure 1A). The ELISA results revealed that mice treated with laminarin exhibited a significantly reduced TNF‐α (about 37%) and IL‐6 (about 29%) in comparison to those mice that only received LPS (Figure 1B,C). Additionally, we measured the levels of TNF‐α and IL‐6 in the serum of LPS‐treated mice from day 1 to day 5. The results showed that laminarin significantly reduced the serum levels of TNF‐α and IL‐6 in LPS‐treated mice on days 1 and 2 (Figure S2). This suggests that laminarin plays a beneficial role in the prognosis of sepsis. In mice with acute lung injury, the lungs exhibited severe inflammation and noticeable morphological changes. However, laminarin reduced the ratio of wet‐to‐dry weight in the lungs and ameliorated the histological changes, which included alveolar infiltration, thickening of alveolar walls, patchy haemorrhage and interstitial edema (Figure 1D,E). Additionally, we measured the protein concentration in the bronchoalveolar lavage fluid (BALF) of mice. The results indicated that laminarin reduced the protein concentration in the BALF of LPS‐induced mice (Figure 1F).

### 3.2 Laminarin Reduced the Level of Inflammation in the Lungs of Septic Mice and in RAW264.7 Cells

**FIGURE 2:**
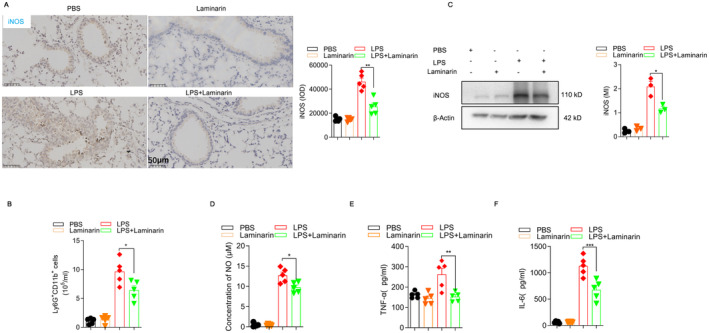
Laminarin reduced the level of inflammation in the lungs of septic mice and in RAW264.7 cells. (A) iNOS protein expression in mouse lung tissue was determined by immunohistochemistry, along with corresponding statistical charts. Images were captured by microscope and analysed as integrated optical density by Image Pro Plus (*n* = 5). (B) Flow cytometry was used to quantify the number of Ly6G^+^ CD11b^+^ neutrophiolseutrophils in the BALF of mice, along with corresponding statistical charts (*n* = 5). (C) iNOS expression level in RAW264.7 cells was determined by western blot analysis, with data shown as mean density normalised by β‐Actin (*n* = 5). (D) Production of NO in RAW264.7 cells was analysed by Griess reagent kit (*n* = 5). (E) Concentration of TNF‐α in culture supernatant was analysed by ELISA (*n* = 5). (F) Concentration of IL‐6 in culture supernatant was analysed by ELISA (*n* = 5). Data are presented as mean ± SD and analysed by one‐way ANOVA. All experiments were repeated thrice. *p* < 0.05 was considered a significant difference between LPS group and LPS + laminarin group; *p < 0.05; **p < 0.01; ***p < 0.001. Data are representative of three independent experiments.

Immune inflammation is a crucial characteristic of LPS‐induced lung injury in mice. Therefore, we investigated the impact of laminarin on the inflammatory levels in lung tissues and macrophages of the LPS‐induced mouse lung injury model. Results indicate that laminarin treatment reduces the expression of pulmonary iNOS in the LPS‐induced mouse model (Figure 2A). During pulmonary inflammation, leukocytes, especially neutrophils, are the first responding immune cells that release ROS, thereby inducing oxidative stress in lung epithelial cells, which contributes to epithelial cell death [21]. We further examined the number of neutrophils in the BALF of mice. The results showed that laminarin treatment decreases the number of neutrophils in the BALF of LPS‐treated mice (Figure 2B).

To investigate the effect of laminarin on macrophages in vitro, we selected RAW264.7 cells, which are commonly used as inflammatory cell models. First, a CCK‐8 assay was performed to determine the optimal working concentration of laminarin for RAW264.7 cells in vitro, which was found to be 0.3125 mg/mL (Figure S3). We further examined the expression of iNOS in RAW264.7 cells using the Western Blot method and found that laminarin effectively inhibits the LPS‐induced expression of iNOS in macrophages (Figure 2C). We further investigated the impact of laminarin on the nitric oxide levels in LPS‐stimulated RAW264.7 cells. The results indicate that laminarin can also reduce the production of nitric oxide in LPS‐stimulated RAW264.7 cells (Figure 2D). We also examined the impact of laminarin on the secretion of inflammatory factors in LPS‐stimulated RAW264.7 cells. The results indicate that laminarin treatment not only reduces the secretion levels of inflammatory factors, specifically TNF‐α, in LPS‐stimulated RAW264.7 cells but also decreases the secretion of IL‐6 (Figure 2E,F).

### 3.3 Laminarin Inhibits the Polarisation of Macrophages Towards M1 Under the Challenge of LPS In Vivo and In Vitro

**FIGURE 3:**
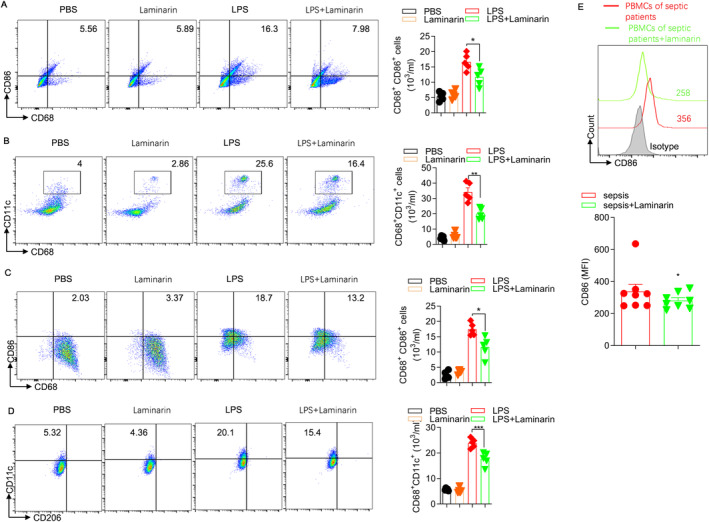
Laminarin inhibits the polarisation of macrophages towards M1 under the challenge of LPS in vivo and in vitro. (A) Flow cytometry was used to detect CD68^+^CD86^+^ M1 macrophages in the BALF of mice, along with corresponding statistical charts (*n* = 5). (B) Flow cytometry was used to detect CD68^+^CD11c^+^ M1 macrophages in the BALF of mice, along with corresponding statistical charts (*n* = 5). (C) Flow cytometry was used to detect CD68^+^CD86^+^ M1 RAW264.7 cells, along with corresponding statistical charts (*n* = 5). (D) Flow cytometry was used to detect CD68^+^CD11c^+^ M1 RAW264.7 cells, along with corresponding statistical charts (*n* = 5). (E) PBMCs were isolated from patients with sepsis and incubated with PBS or 0.3125 mg/mL laminarin (*n* = 8). The expression level of CD86 in PBMCs treated with PBS (shown in red peak) or laminarin (shown in green peak) was determined by flow cytometry. Mean fluorescence intensity (MFI) was presented as the expression level of CD86. Data are presented as mean ± SD and analysed by two‐tailed Student's *t* test. All images were displayed by FlowJo software. *p* < 0.05 was considered a significant difference between LPS group and LPS + laminarin group; *p < 0.05; **p < 0.01; ***p < 0.001. Data are representative of three independent experiments.

Macrophages are the crucial cell population in mediating inflammation and tissue damage and serve as a key cellular target for the treatment of ALI. During the development of sepsis, there was a significant increase in the number of M1 macrophages, which contribute to acute lung injury. To explore whether laminarin improved acute lung injury by inhibiting the polarisation of M1 macrophages, the markers of M1 macrophages in the lungs of septic mice with acute lung injury were assessed using flow cytometry. In the lungs of mice, CD68 was used to label macrophages, whereas CD86 was employed to mark M1 macrophages. The percentage of CD68^+^CD86^+^ cells was considered as the percentage of M1 macrophages. In the presence of LPS treatment, the percentage of M1 macrophages increased significantly, nearly doubling when compared to the PBS group. However, when laminarin was administered, the polarisation of M1 macrophages was effectively inhibited. The number of CD68^+^CD86^+^ cells decreased by two‐thirds compared to the LPS group, approaching levels similar to those observed in the PBS group (Figure 3A). We used another marker, CD11c, to identify M1 macrophages. The results also demonstrated that laminarin inhibits the polarisation of LPS‐treated macrophages towards the M1 phenotype (Figure 3B).

To examine the impact of laminarin on macrophages in vitro, we selected RAW264.7 cells, a type of cell commonly used as models for inflammatory responses. Initially, a CCK‐8 assay was performed to determine the optimal working concentration of laminarin for RAW264.7 cells in vitro, which was found to be 0.3125 mg/mL (Figure S2). Similarly, laminarin significantly reduced the percentage of CD68^+^CD86^+^ RAW264.7 cells that were stimulated with LPS by more than 15% (Figure 3C). The results using CD11c as an M1 marker were also consistent (Figure 3D). Furthermore, peripheral blood mononuclear cells (PBMCs) obtained from sepsis patients (*n* = 8) were isolated and cultured with 0.3125 mg/mL of laminarin for 24 h. Subsequently, flow cytometry analysis was carried out to assess the levels of CD86. The results demonstrated that, in comparison to the control group, the administration of laminarin led to a reduction in the CD86 levels in sepsis patients (Figure 3E).

### 3.4 Laminarin Inhibited the Uptake of Glucose and the Production of Lactate in M1 Macrophages

**FIGURE 4:**
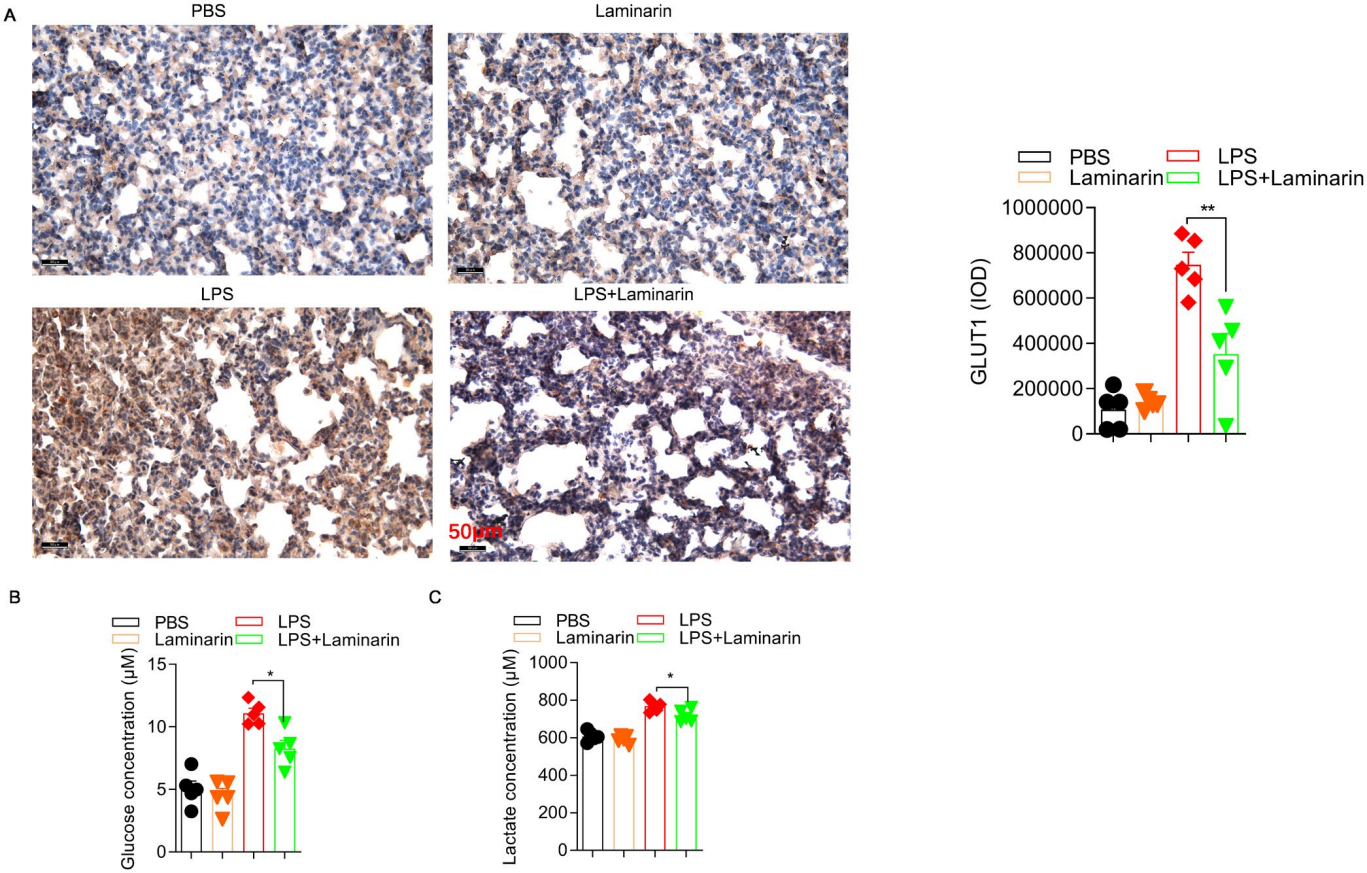
Laminarin inhibited the uptake of glucose and production of lactate in M1 macrophages. (A) Glucose uptake in the lungs of LPS‐induced septic mice was shown as Glut1 expression, determined by immunohistochemistry. Images were captured by a microscope and analysed as integrated optical density by Image Pro Plus (scale bar magnification = 50 μm) (*n* = 5). (B) RAW264.7 cells were harvested and lysed; the supernatant was collected for glucose concentration analysis by WST reaction (*n* = 5). (C) Lactate production of RAW264.7 cells was assayed by determining lactate concentration in the supernatant with WST reaction (*n* = 5). Data are presented as mean ± SD and analysed by one‐way ANOVA. *p* < 0.05 was considered a significant difference between the LPS group and the LPS + laminarin group; *p < 0.05; **p < 0.01. Data are representative of three independent experiments.

Glycolysis is the primary metabolic pathway of M1 macrophages and is characterised by the rapid consumption of glucose. Glucose serves as the substrate for glycolysis, and the enzyme hexokinase catalyses the conversion of glucose into glucose 6‐phosphate. Consequently, glucose uptake is a critical step in the glycolytic pathway. In the lungs of mice, the expression of glucose transporter 1 (Glut1) was assessed through immunohistochemistry (IHC). In comparison to the PBS group, mice that received LPS exhibited a heightened level of Glut1 expression, indicating an increase in glucose uptake. However, the administration of laminarin significantly reduced the expression of Glut1 (Figure 4A). To directly investigate the effect of laminarin on glucose uptake in macrophages, ELISA assay was conducted to measure the glucose concentration in RAW264.7 cells. The results indicated that, when stimulated with LPS, the addition of laminarin reduced the concentration of glucose by approximately 30% in RAW264.7 cells (Figure 4B). Lactate is one of the products of glycolysis. ELISA analysis was employed to measure the concentration of lactate in the supernatant of RAW264.7 cells. As depicted in Figure 4C, there was no significant difference between the laminarin‐alone group and the PBS group. However, a significant decrease in lactate concentration was observed in the presence of laminarin when challenged with LPS.

### 3.5 Laminarin Treatment Reduced ROS Levels and Downregulated NLRP3 Inflammasome in LPS‐Stimulated Macrophages

**FIGURE 5:**
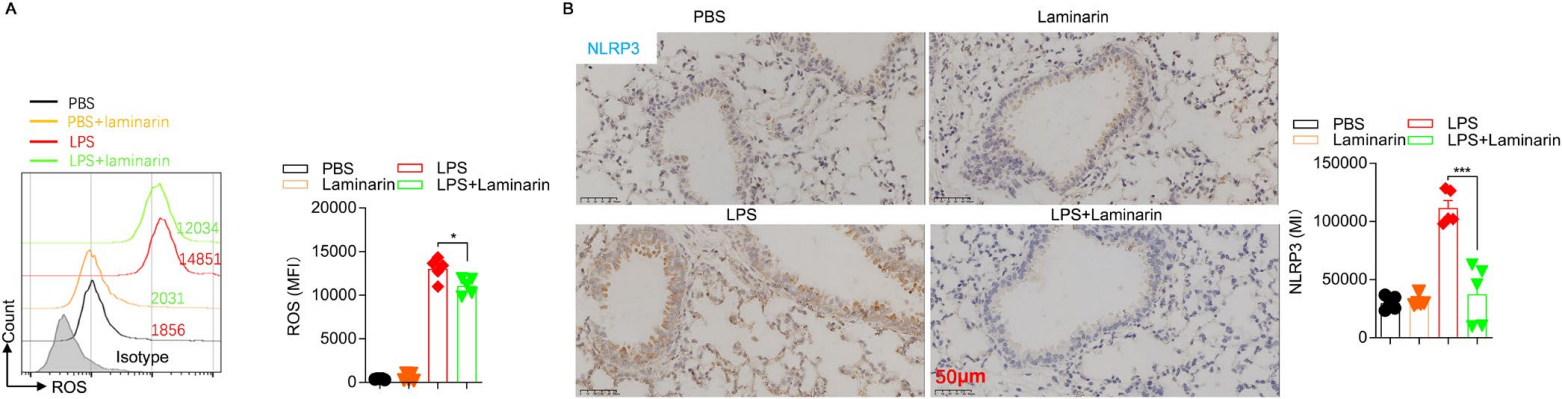
Laminarin treatment reduced ROS levels and downregulated the expression of the inflammatory molecule NLRP3 in LPS‐stimulated macrophages. (A) RAW264.7 cells were harvested and loaded with DCFH‐DA. After incubation, the cells were washed with DMEM to remove unloaded DCFH‐DA. DCF fluorescence intensity was measured by flow cytometry (*n* = 5). (B) NLRP3 protein expression in lung tissue was determined by immunohistochemistry (*n* = 5). Images were captured by a microscope and analysed as integrated optical density by Image Pro Plus (scale bar magnification = 50 μm). Data are presented as mean ± SD and analysed by one‐way ANOVA. *p* < 0.05 was considered a significant difference between the LPS group and the LPS + laminarin group; *p < 0.05; ***p < 0.001. Data are representative of three independent experiments.

Reactive Oxygen Species (ROS) is a key indicator of cellular oxidative stress and a trigger of inflammation in the body. We found that laminarin treatment significantly reduced ROS levels in LPS‐stimulated macrophages (Figure 5A). Further investigation of inflammatory signalling pathways in macrophages revealed significant downregulation of NLRP3 inflammasome expression upon laminarin treatment (Figure 5B). This suggests that laminarin treatment inhibits NLRP3‐mediated inflammation.

### 3.6 Laminarin May Potentially Inhibit Macrophage M1 Polarisation by Influencing the HIF‐1α Signalling Pathway

**FIGURE 6:**
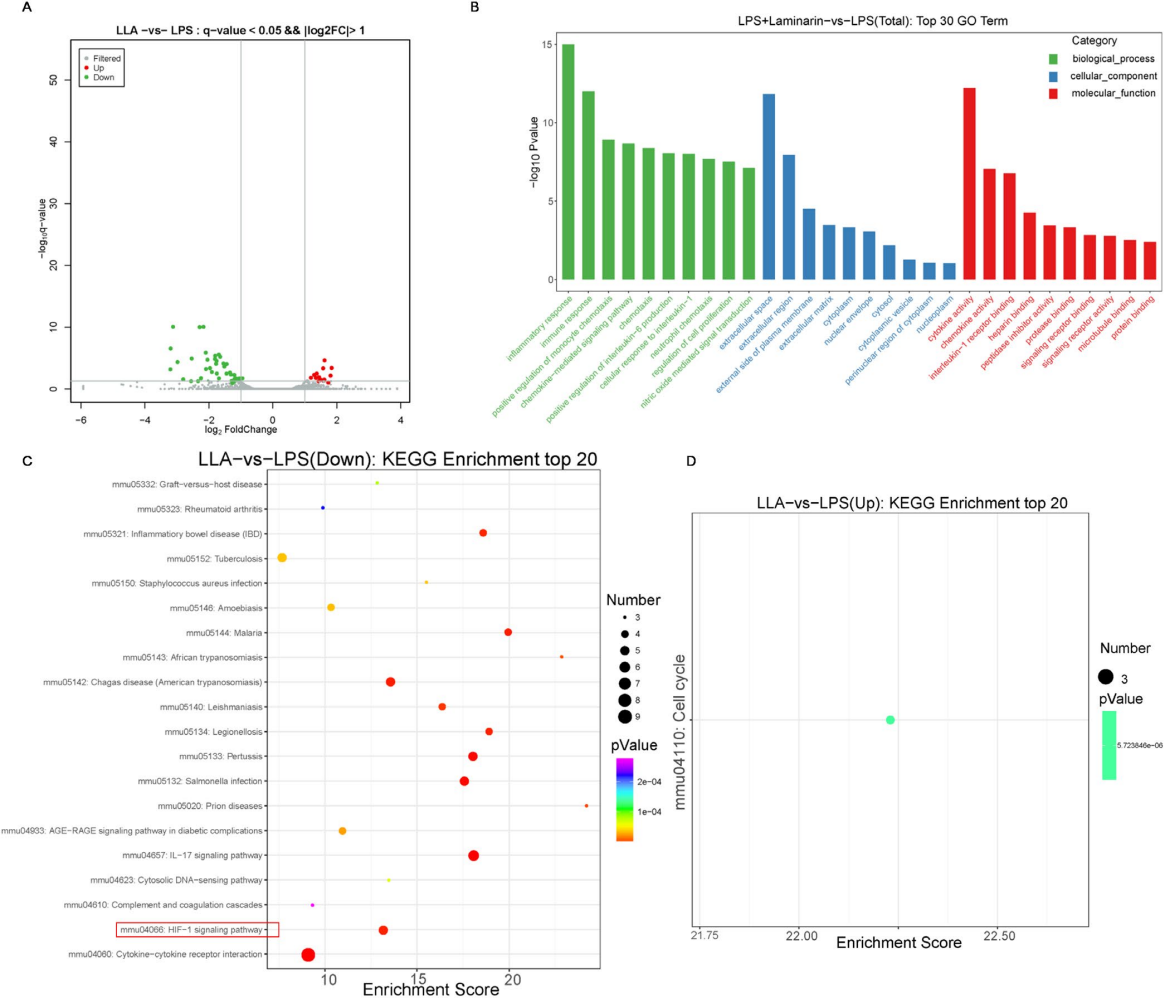
Laminarin may potentially inhibit macrophage M1 polarisation by downregulating the HIF‐1α signalling pathway. (A) Differentially expressed genes between the LPS + laminarin group and the LPS group. Each dot represents a single gene, with *q* < 0.05 and |log2FC| > 1 considered as significantly differentially expressed genes. The horizontal and vertical coordinates represent the log2FC values of the biological replicate multiple differences between the LPS + laminarin group and the LPS group. Red indicates significantly upregulated genes, dark blue indicates significantly downregulated genes, and grey indicates non‐significant differences (*n* = 3). (B) GO enrichment analysis of differentially expressed genes in biological processes (green columns), cellular components (blue columns), and molecular functions (red columns) between the LPS + laminarin group and the LPS group. The horizontal and vertical coordinates represent the GO term and −log10 *p*‐value, respectively (*n* = 3). (C) KEGG pathway enrichment analysis showing significantly upregulated pathways (*n* = 3). (D) KEGG pathway enrichment analysis showing significantly downregulated pathways (*n* = 3). The vertical coordinates represent the pathway name. The horizontal axis is the enrichment score, and the size of the bubbles indicates the number of differentially expressed genes related to the same pathway.

To investigate the potential pathways involved in laminarin's inhibition of M1 macrophage polarisation, RNA sequencing analysis was conducted on RAW264.7 cells. Genes with values of *q* < 0.05 and |log2FC| > 1 were considered significantly differentially expressed between the LPS + laminarin group and the LPS group. Compared to RAW264.7 cells induced by LPS, laminarin downregulated 45 genes (illustrated as green dots) and upregulated 14 genes (indicated as red dots) (Figure 6A). These genes were associated with inflammatory response and immune response in biological processes, as well as extracellular space and extracellular region in cellular components, along with cytokine activity and chemokine activity in molecular functions (Figure 6B).

Subsequently, KEGG enrichment analysis of the differentially expressed genes was conducted. The results showed that laminarin downregulated genes related to the IL‐17 signalling pathway, HIF‐1α signalling pathway, and the cytosolic‐DNA sensing pathway, while it upregulated genes associated with the cell cycle pathway (Figure 6C,D). Since HIF‐1α has been demonstrated to promote glycolysis in LPS‐induced acute lung injury, it is speculated that laminarin may potentially inhibit macrophage M1 polarisation by influencing the HIF‐1α signalling pathway.

### 3.7 Laminarin Inhibited M1 Macrophage Polarisation by Downregulating the HIF‐1α Pathway

**FIGURE 7:**
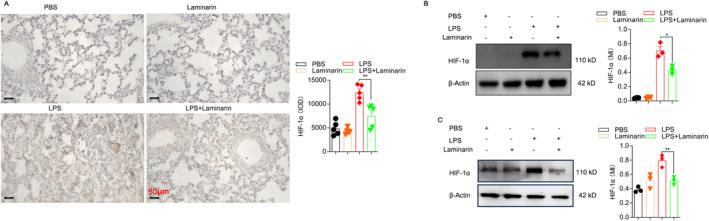
Laminarin inhibited M1 macrophage polarisation by downregulating the HIF‐1α pathway. (A) HIF‐1α expression level in lung tissue was determined by immunohistochemistry (*n* = 5). Images were captured by a microscope and analysed as integrated optical density using Image Pro Plus. (B) HIF‐1α expression level in lung tissue was determined by western blot analysis, with data shown as mean density normalised by β‐Actin (*n* = 3). (C) HIF‐1α expression level in RAW264.7 cells was determined by western blot analysis, with data shown as mean density normalised by β‐Actin (*n* = 3). Data are presented as mean ± SD and analysed by one‐way ANOVA. *p* < 0.05 was considered a significant difference between the LPS group and the LPS + laminarin group; *p < 0.05; **p < 0.01. Data are representative of three independent experiments.

We further validated the expression of key molecules in the HIF‐1α signalling pathway through experimental verification. Results indicated that laminarin treatment successfully reduced the expression of HIF‐1α in the lungs of septic mice and in LPS‐stimulated RAW264.7 cells (Figure 7A,B).

**FIGURE 8:**
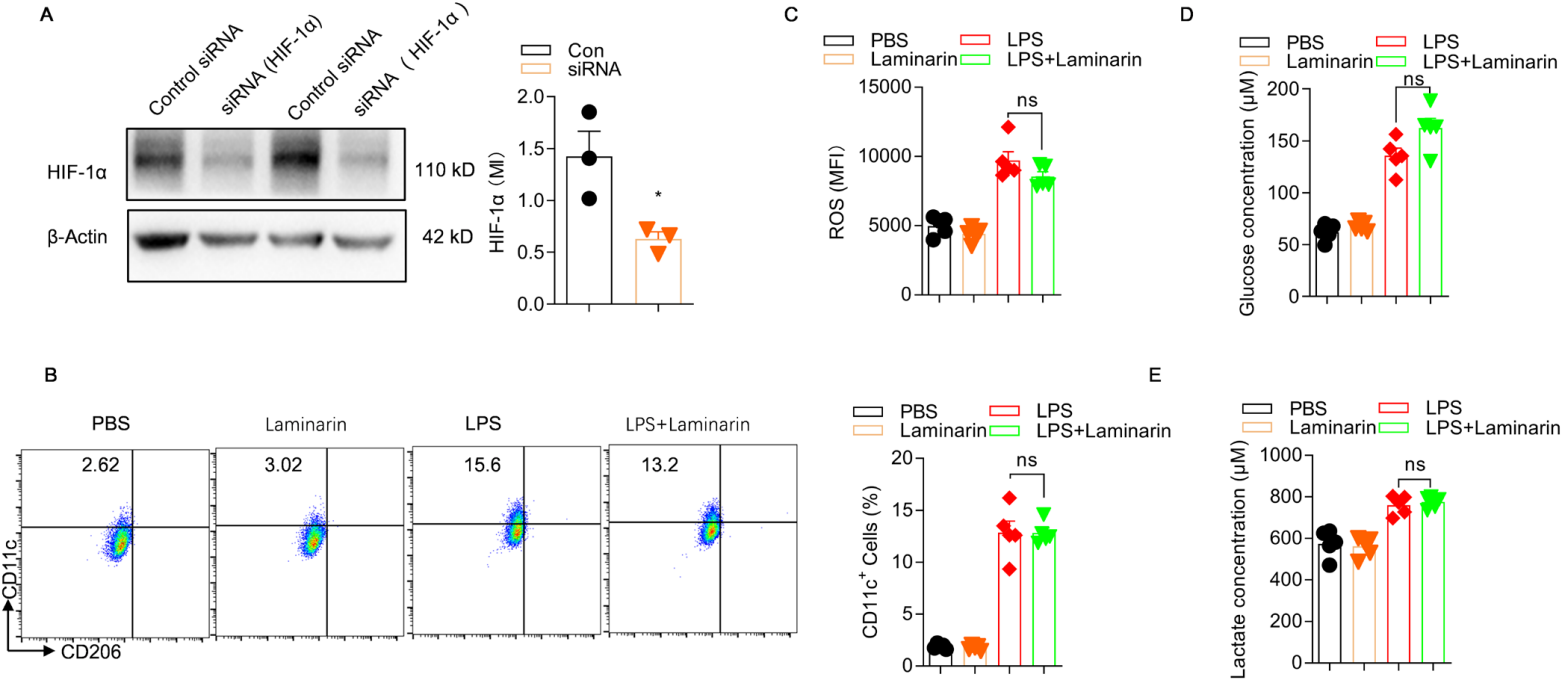
After knocking down HIF‐1α, the inhibitory effects of laminarin on ROS production, glucose uptake and lactate production in LPS‐treated macrophages were abolished. (A) After transfecting RAW 264.7 cells with HIF‐1α siRNA, the knockdown efficiency was assessed using Western blot analysis (*n* = 3). (B) After silencing HIF‐1α, the proportion of CD11c+ M1 cells was detected. (C) After silencing HIF‐1α, the levels of ROS production in RAW 264.7 cells for each treatment group were assessed using flow cytometry (*n* = 5). (D) After silencing HIF‐1α, glucose levels in RAW 264.7 cells from each treatment group were measured using a glucose assay kit (*n* = 5). (E) After silencing HIF‐1α, lactate levels in RAW 264.7 cells from each treatment group were measured using a lactate assay kit (*n* = 5). Data were presented as mean ± SD and analysed by one‐way ANOVA. *p* < 0.05 was considered a significant difference between the LPS group and the LPS + laminarin group; *p < 0.05. Data are representative of three independent experiments.

After knocking down HIF‐1α in RAW264.7 cells using siRNA (Figure 8A), we found no significant difference in the proportion of CD11c^+^ M1 cells between the group treated with LPS alone and the group treated with LPS + laminarin (Figure 8B). Consistent with these findings, knocking down HIF‐1α in RAW264.7 cells resulted in no significant differences in ROS production, intracellular glucose levels or lactate levels between the group treated with LPS alone and the group treated with LPS + laminarin (Figure 8C–E).

## 4 Discussion

Sepsis is a severe condition that carries a high mortality rate, and It can lead to multiple organ failure and is also a common factor in inducing ARDS [20, 22]. According to relevant epidemiological studies, the incidence of ALI in sepsis patients is 68.2%, and the 90‐day mortality rate for patients with combined ALI is as high as 35.5% [21]. Currently, many interventions for the treatment of ALI/ARDS, such as the use of dexamethasone and low tidal volume ventilation [23], have not improved survival rates in patients. In our study, we found that the anti‐inflammatory effects of laminarin can be utilised to alleviate ALI.

In recent years, it has been reported that laminarin [24], the primary bioactive component of seaweed characterised by its 1,3‐β‐glucan with 1,6‐β‐glucose branches, has shown promise in the treatment of conditions such as arthritis, pneumonia, and tumors [25]. However, the specific effects and mechanisms of laminarin in the context of acute lung injury in sepsis remain unknown. In this study, we have demonstrated, for the first time, that laminarin effectively improves acute lung injury induced by lipopolysaccharide (LPS) by inhibiting the macrophage M1 polarisation.

In this research, a septic mouse model was established using Gram‐negative bacterial LPS, And after adjusting the dosage of LPS to an appropriate level, we found that sepsis can further induce ALI. Laminarin was administered 1 h after the injection of LPS. While there has been limited research on laminarin in the context of sepsis, this study demonstrated that laminarin was effective in improving pulmonary edema, increasing the survival rate of mice induced by LPS, and inhibiting the levels of IL‐6, a biomarker of sepsis, in the serum of septic mice [26, 27]. While previous studies, such as Reynolds et al., have indicated that the gene expression of IL‐6 in macrophages can be inhibited by laminarin, it is important to note that there has been limited data demonstrating the effects of laminarin on IL‐6 in the context of sepsis. This study contributes to our understanding of the potential impact of laminarin on IL‐6 levels in sepsis [28]. The results of this study provide evidence that laminarin effectively and directly improves acute lung injury in septic mice.

In acute lung injury (ALI) induced by LPS, various cells such as macrophages, neutrophils, dendritic cells (DCs), and natural killer (NK) cells are involved in its development [29]. Among these, neutrophils and macrophages are two key innate immune cells involved in ALI. The influx of neutrophils into the lungs is a hallmark of ALI [14], and neutrophils play a crucial role in the early stages of ALI, rapidly responding and participating in the clearance of pathogens [30]. Studies have shown [31] that the number of neutrophils increases in ALI, and the proportion and distribution of their subsets are closely related to the occurrence and progression of ALI. The death of neutrophils is also an important cause of lung injury, such as the formation of neutrophil extracellular traps (NETs), ferroptosis, pyroptosis, apoptosis, autophagy, necrosis and necroptosis [30]. In acute inflammatory models, macrophages not only regulate the body's inflammatory response but also exacerbate inflammation by secreting various cytokines such as tumour necrosis factor α (TNF‐α), interleukin 1β (IL‐1β) and IL‐6 [32]. In addition, macrophages play an important role in promoting tissue repair and immune regulation. They can polarise into M1 (pro‐inflammatory) and M2 (anti‐inflammatory/reparative) phenotypes in response to different local microenvironmental stimuli [33]. In the inflammatory response of ALI, macrophages tend to polarise towards the M1 phenotype, leading to exacerbated lung inflammation, and later, they can transform into the M2 phenotype, promoting tissue repair and regeneration [34].

In the course of sepsis development, the balance between M1 and M2 macrophages is disrupted, leading to a substantial increase in M1 macrophages. As a result, interventions that inhibit the polarisation of M1 macrophages hold promise for the treatment of acute lung injury in sepsis. Previous studies have indicated that β‐glucans, such as laminarin, play a role in the regulation of macrophage polarisation [35]. Laminarin is a soluble 1,3‐β‐glucan with 1,6‐β‐glucose branches, and it functions as an antagonist of Dendritic cell‐associated C‐type lectin‐1 (Dectin‐1) [36]. While the specific role of laminarin on M1 macrophages may not have been extensively reported in previous studies, it is worth noting that in some research, Dectin‐1 has been identified as a marker for M2 macrophages [37] and necessary for activation for M1 macrophages and induction of pro‐inflammatory cytokines [38, 39]. Additionally, research by Correia et al. has shown that Zymosan, an agonist of Dectin‐1, can increase the expression of CD80 and CD86, which are markers associated with M1 macrophages [40]. Based on the findings from these research studies, it was hypothesised that laminarin improves acute lung injury in sepsis by inhibiting the polarisation of M1 macrophages. In this research, the administration of laminarin resulted in a significant decrease in the expression of CD86, a marker associated with M1 macrophages, in the lungs of septic mice, LPS‐induced RAW264.7 cells, and PBMCs from patients with sepsis. This suggests that laminarin effectively inhibits M1 macrophage polarisation. In bronchoalveolar lavage fluid, an increase in the number of neutrophils can be observed. However, we have also observed that with the use of laminarin in the context of acute lung injury caused by sepsis, there is a reduction in the number of neutrophils, while the pro‐inflammatory M1 macrophages decrease and the anti‐inflammatory M2 macrophages increase. In the RAW264.7 cell line, we have also found that laminarin can inhibit the M1 polarisation of macrophages. Although both neutrophils and macrophages undergo changes after the use of laminarin, the increase and decrease in the number of neutrophils are closely related to the severity of inflammation, so it often serves as an evaluation indicator in acute lung injury models. We believe that the reduction in their numbers after using laminarin may be due to the decrease in pro‐inflammatory factors secreted by macrophages, leading to reduced neutrophil recruitment, rather than laminarin directly causing a decrease in neutrophils. The anti‐inflammatory effects of laminarin, especially its inhibitory effect on the M1 polarisation of macrophages, draw our attention to the key role of macrophages in alleviating LPS‐induced acute lung injury by laminarin.

Nitric oxide (NO) produced by M1 macrophages can promote the secretion of inflammatory cytokines and exacerbate lung injury in sepsis. The results demonstrate that laminarin significantly reduced the concentration of NO in RAW264.7 cells stimulated by LPS, which is consistent with findings reported in previous literature by Ng [41]. In in vivo experiments, the expression level of NO was assessed by measuring the expression of nitric oxide synthase. iNOS is known to be activated in M1 macrophages [42]. Following the administration of eNOS small interfering RNA (siRNA), the expression of M1 markers decreased while the expression of M2 markers increased in THP‐1 cells [43]. In an acute lung injury mice model, laminarin was demonstrated to effectively reduce the expression of iNOS but did not significantly increase the expression of eNOS. This suggests that laminarin decreases the production of NO by inhibiting iNOS.

Glycolysis, the primary metabolic pathway of M1 macrophages, plays a pivotal role in the progression of sepsis. It is a key factor that can exacerbate the severity of this condition [44]. During the process of glycolysis in macrophages, there is rapid consumption of glucose, and this metabolic pathway results in the overproduction of lactate [45, 46]. In the presence of LPS challenges, the expression level of Glut1 in the lungs of mice increased, and there was an elevation in the concentration of glucose and lactate in RAW264.7 cells. However, under the administration of laminarin, this trend was reversed. These pieces of evidence collectively indicate that laminarin effectively inhibits the uptake of glucose by macrophages.

To explore the potential mechanisms underlying the regulation of macrophage polarisation, RNA sequence analysis was conducted. The results suggested that laminarin may inhibit macrophage M1 polarisation via the HIF‐1α pathway. This hypothesis was derived from the enrichment analysis of differently expressed genes in GO analysis and KEGG analysis. It is worth noting that there have been limited reports about the effects of laminarin on HIF‐1α. However, the results of this study have shown that laminarin significantly decreases HIF‐1α expression, both in vivo and in vitro. Overexpression of ROS has been implicated in promoting the production of hypoxia‐HIF‐1α in the context of acute lung injury [47, 48]. Indeed, while the study did not directly prove that laminarin reduced HIF‐1α by lowering ROS levels, it provided evidence that laminarin inhibits macrophage M1 polarisation by targeting the HIF‐1α pathway. NLRP3 is a protein associated with inflammation and is known to play a role in various inflammatory processes. Recent research has suggested that NLRP3 is involved in the glycolytic metabolism associated with acute lung injury. Inhibition of glycolysis, such as with 2‐DG, has been shown to significantly decrease the expression of NLRP3 in mice with acute lung injury [12]. Furthermore, a study by Guan et al. reported that the inhibition of HIF‐1α led to a failure in the activation of NLRP3. In this study, the administration of laminarin was found to inhibit the activation of the NLRP3 inflammasome induced by LPS [42]. In this study, we observed that knocking down HIF‐1α in RAW 264.7 cells abolished the inhibitory effects of laminarin on ROS and lactate production. This result supports the notion that laminarin inhibits glycolysis through the HIF‐1α pathway, to some extent, contributing to the regulation of NLRP3 inflammasome activation.

## 5 Conclusions

In summary, this study has identified laminarin as a promising novel candidate drug for the treatment of acute lung injury induced by sepsis.

## Author Contributions

**Liming Zeng:** conceptualization (equal). **Jieyu Zhang:** data curation (equal). **Rongrong Song:** formal analysis (equal). **Xinhuai Dong:** formal analysis (equal), investigation (equal), methodology (equal). **Zibo Wei:** data curation (equal). **Xiaoyan Li:** formal analysis (equal). **Xiaokang Zeng:** investigation (equal). **Jie Yao:** funding acquisition (equal), project administration (equal).

## Ethics Statement

The protocol of the study was approved by the Ethics Review Committee of Shunde Hospital, Southern Medical University, Foshan, Guangdong, China (KYLS20231109). The animal experiment protocol was approved by the Animal Ethics Committee of Southern Medical University (No. SDYY‐YJ‐2205‐002).

## Consent

All authors have agreed to publish this manuscript.

## Conflicts of Interest

The authors declare no conflicts of interest.

## Supporting information


**Figure S1.** Septic mice model induced by different concentrations of LPS. Male C57BL6/J mice aged 8–12 weeks were intraperitoneally injected with varying doses of lipopolysaccharide (LPS) (0, 15, 25, 35 mg/kg; *n* = 3 per group). After 6 h, mice were euthanized, and lung tissues were collected for haematoxylin–eosin staining. Microscopic images were captured (Scale bar: 100 μm). Parameters such as bleeding (indicated by black arrows), inflammatory cell infiltration, alveolar disorganisation and alveolar wall thickness were evaluated to assess lung injury severity in LPS‐treated mice compared to those receiving PBS (*n* = 5).

**Figure S2.** TNF‐α and IL‐6 levels in serum from days 1 to 5 in mice treated with LPS or LPS + laminarin. (A) Serum TNF‐α levels from days 1 to 5 in mice treated with LPS or LPS + laminarin. (B) Serum IL‐6 levels from days 1 to 5 in mice treated with LPS or LPS + laminarin (*n* = 5). Data are presented as mean ± SD and were analysed by one‐way ANOVA; *p* < 0.05 was considered significant (*p < 0.05) between the LPS group and LPS + laminarin group.

**Figure S3.** Viability of RAW264.7 cells after incubation at different concentrations of laminarin. (A) Viability (%) of RAW264.7 cells after 24 h of incubation with laminarin at concentrations ranging from 5 mg/mL to 0 mg/mL (*n* = 5). (B) Viability (%) of RAW264.7 cells after 48 h of incubation with laminarin at concentrations ranging from 5 mg/mL to 0 mg/mL (*n* = 3). Cell viability was assessed using the CCK‐8 assay. Data are presented as mean ± SD and were analysed by one‐way ANOVA. Significant differences were considered at *p* < 0.05 (*), *p* < 0.01 (**), and *p* < 0.001 (***), compared to the 0 mg/mL laminarin control.

## Data Availability

The data used to support the findings of this study are available from the corresponding author upon request.
