# Supplementary figures and images for: Laminarin Alleviates Acute Lung Injury Induced by LPS Through Inhibition of M1 Macrophage Polarisation
Source: J Cell Mol Med. 2025 Mar 5;29(5):e70440. doi: 10.1111/jcmm.70440 (PMC11882389; doi:10.1111/jcmm.70440)

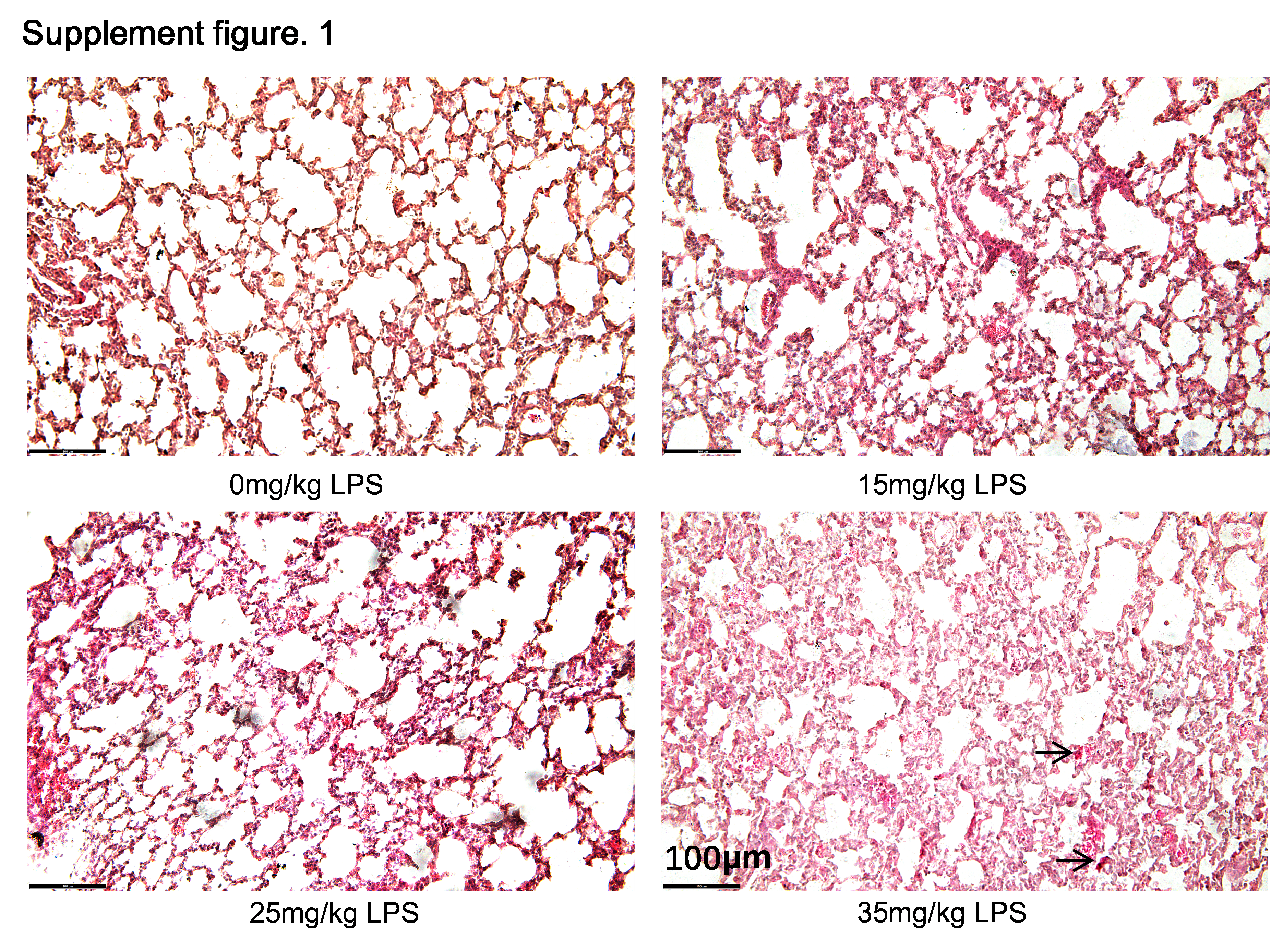

Supplement: Supplementary file 1 — Figure S1. Septic mice model induced by different concentrations of LPS. Male C57BL6/J mice aged 8–12 weeks were intraperitoneally injected with varying doses of lipopolysaccharide (LPS) (0, 15, 25, 35 mg/kg; n = 3 per group). After 6 h, mice were euthanized, and lung tissues were collected for haematoxylin–eosin staining. Microscopic images were captured (Scale bar: 100 μm). Parameters such as bleeding (indicated by black arrows), inflammatory cell infiltration, alveolar disorganisation and alveolar wall thickness were evaluated to assess lung injury severity in LPS‐treated mice compared to those receiving PBS (n = 5). [file JCMM-29-e70440-s002.tif]

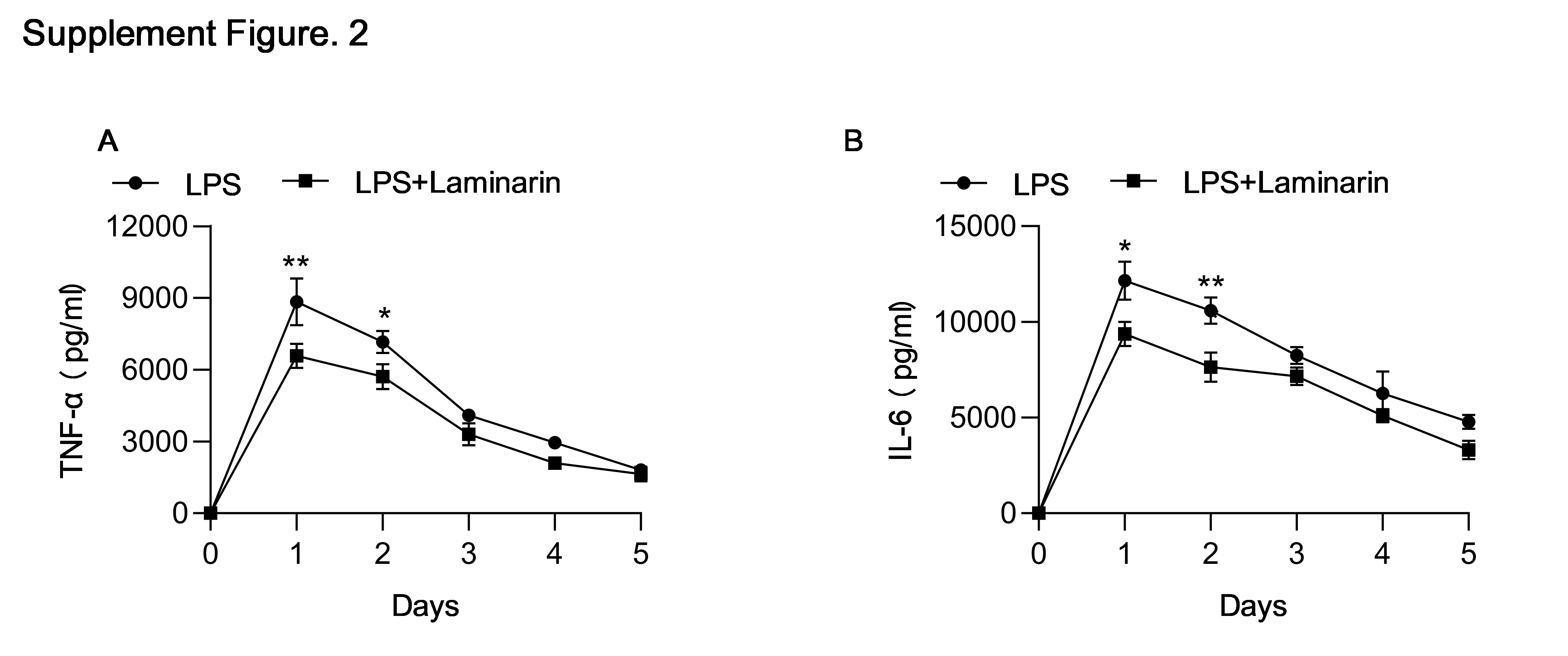

Supplement: Supplementary file 2 — Figure S2. TNF‐α and IL‐6 levels in serum from days 1 to 5 in mice treated with LPS or LPS + laminarin. (A) Serum TNF‐α levels from days 1 to 5 in mice treated with LPS or LPS + laminarin. (B) Serum IL‐6 levels from days 1 to 5 in mice treated with LPS or LPS + laminarin (n = 5). Data are presented as mean ± SD and were analysed by one‐way ANOVA; p < 0.05 was considered significant (*p < 0.05) between the LPS group and LPS + laminarin group. [file JCMM-29-e70440-s003.tif]

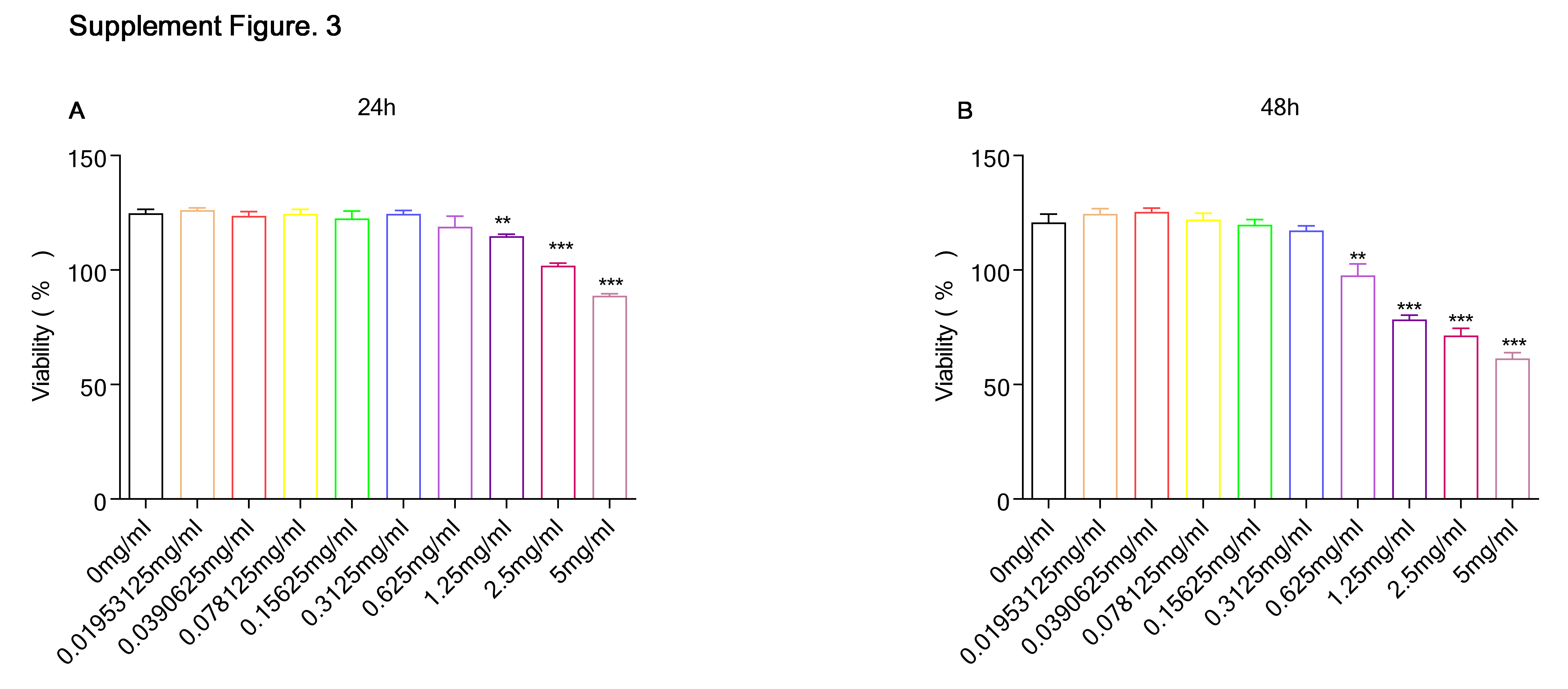

Supplement: Supplementary file 3 — Figure S3. Viability of RAW264.7 cells after incubation at different concentrations of laminarin. (A) Viability (%) of RAW264.7 cells after 24 h of incubation with laminarin at concentrations ranging from 5 mg/mL to 0 mg/mL (n = 5). (B) Viability (%) of RAW264.7 cells after 48 h of incubation with laminarin at concentrations ranging from 5 mg/mL to 0 mg/mL (n = 3). Cell viability was assessed using the CCK‐8 assay. Data are presented as mean ± SD and were analysed by one‐way ANOVA. Significant differences were considered at p < 0.05 (*), p < 0.01 (**), and p < 0.001 (***), compared to the 0 mg/mL laminarin control. [file JCMM-29-e70440-s001.tif]
